# Lipid droplet density alters the early innate immune response to viral infection

**DOI:** 10.1371/journal.pone.0190597

**Published:** 2018-01-02

**Authors:** Ebony A. Monson, Keaton M. Crosse, Mithun Das, Karla J. Helbig

**Affiliations:** Department of Physiology, Anatomy and Microbiology, La Trobe University, Melbourne, Victoria; University of Nebraska-Lincoln, UNITED STATES

## Abstract

The cellular localisation of many innate signalling events following viral infection has yet to be elucidated, however there has been a few cases in which membranes of certain cellular organelles have acted as platforms to these events. Of these, lipid droplets (LDs) have recently been identified as signalling platforms for innate TLR7 and 9 signalling. Despite their wide range of similar roles in various metabolic pathways, LDs have been overlooked as potential platforms for antiviral innate signalling events. This study established an *in vitro* model to evaluate the efficiency of the early innate immune response in cells with reduced LD content to the viral mimics, dsDNA and dsRNA, and Sendai viral infection. Using RT-qPCR, the expression of IFN-β and IFN-λ was quantified following stimulation along with the expression of specific ISGs. Luciferase based assays evaluated the combined expression of ISRE-promoter driven ISGs under IFN-β stimulation. Cellular LD content did not alter the entry of fluorescently labelled viral mimics into cells, but significantly decreased the ability of both Huh-7 and HeLa cells to produce type I and III IFN, as well as downstream ISG expression, indicative of an impeded innate immune response. This observation was also seen during Sendai virus infection of HeLa cells, where both control and LD reduced cells replicated the virus to the same level, but a significantly impaired type I and III IFN response was observed in the LD reduced cells. In addition to altered IFN production, cells with reduced LD content exhibited decreased expression of specific antiviral ISGs: Viperin, IFIT-1 and OAS-1 under IFN-β stimulation; However the overall induction of the ISRE-promoter was not effected. This study implicates a role for LDs in an efficient early innate host response to viral infection and future work will endeavour to determine the precise role these important organelles play in induction of an antiviral response.

## Introduction

The innate immune response constitutes the first line of host defence to invading viruses; as such, viral infection of a mammalian cell triggers the activation of a number of pattern recognition receptors (PRRs), with subsequent pathway activation resulting in the production of interferon (IFN). IFNs are secreted cytokines, released into the extracellular milieu where they act in both an autocrine and a paracrine manner, binding to specific receptors on the surface of infected and uninfected cells [1]. The activation of a secondary signalling pathway, the JAK/STAT pathway, initiates the expression of hundreds of interferon stimulated genes (ISGs). It is these ISGs which promote an antiviral state, decreasing the susceptibility of uninfected cells to subsequent infection by impeding viral proliferation [1]. The germline-encoded innate immune system is not only able to detect and neutralise incoming foreign pathogens but it also primes and shapes the adaptive immune response [2].

The localisation of many of the key adaptor molecules within the PRR or JAK/STAT signalling pathways remains elusive, although a defining feature of eukaryotic cells is the use of membrane-bound organelles to compartmentalize activities and serve as scaffolds for signal transduction [3]. Signalling organelles have been hypothesised as the site where activation of key adaptor molecules occurs, and have been shown to dictate the intensity and/or speed of innate signalling pathway activation [3, 4]. The mitochondria, peroxisome, endoplasmic reticulum and the mitochondrial associated membranes (MAM) are all organelles that have been implicated in the coordination of host signalling events, and have only recently been demonstrated to play a role in the control of antiviral immunity, and provide a platform for signalling events (As Reviewed in [5]).

The role of LDs as a signalling platform in the early innate immune response is relatively unexplored. Lipid droplets (LDs) consist of a neutral lipid core, predominantly triglycerides and sterol esters, surrounded by a monolayer of phospholipids and a variety of proteins (Reviewed in [6]. The roles of LDs as a signalling platform are best described for lipid storage, however they have been implicated in a wide range of other functions, including acting as signalling platforms in lipid mobilization, vesicular trafficking, protein folding, protein storage and autophagy [7–11]. Recently, LDs in mammalian immune cells, such as neutrophils and macrophages have been shown to play important roles in inflammatory or infectious processes, increasing in number upon different types of immune challenges and thereby serving as reliable markers of immune cell activation [12]. Similarly, LD’s have also been shown to accumulate in response to bacterial and viral infection in the mosquito, and have been linked to immune control in this host [13].

LDs have been demonstrated previously, to play a critical role in the host antiviral response in the mouse, acting as a platform for the ISG viperin. Viperin is one of the few ISGs shown to have direct antiviral activity in limiting a broad range of viruses (as reviewed in [14]), and this pan-viral protein requires its localisation to the LD to inhibit replication of HCV [15]. Viperin’s localisation to the LD, has also been implicated in efficient TLR7 and TLR9 signalling events [16]. Upon activation of TLR7 and TLR9, Myeloid Differentiation Primary Response 88 (MyD88) mediates signals to the LDs, where viperin interacts with the signal mediators, Interleukin-1 Receptor-Associated Kinase (IRAK) 1 and TNF Receptor-Associated Factor (TRAF) 6. In a stimulation-dependent manner, viperin facilitates K63-linked ubiquitination of IRAK1, and enhances the production of type I and III IFN [16].

Interestingly, the role of the LDs in anti-viral signalling events remains relatively unexplored, therefore this work sought to describe the innate response to viral stimulation in cells with a decreased LD content, to assess the value of these organelles in the transduction of an anti-viral innate immune response.

## Materials and methods

### Cell lines and virus stocks

The human hepatoma cell line Huh-7 and Human epithelial cell line HeLa were originally obtained from the ATCC and were maintained as previously described [17]. Sendai virus was kindly supplied by Dr Ashley Mansell (Monash Institute of Medical Research).

### Lowered serum experiments

Cells were either given low serum media containing 2% FCS, or control serum media containing 10% FCS and were incubated in T75cm^2^ flasks for 48 hours prior to plating at the required cell density. Cell culture media on all experiments was changed 30 minutes prior to the beginning of the experiment, with all transfections and experiments being performed in 10% FCS.

### Real-time polymerase chain reaction

All experiments involving real-time PCR were performed in 12 well plates with Huh-7 and HeLa cells seeded at 7 × 10^4^/well, 24 hours prior to transfection/infection, and performed at least in triplicate. Total RNA was extracted from cells using TriSure reagent (Bioline). First strand cDNA was synthesized from total RNA and reverse transcribed using a Tetro cDNA synthesis kit (Bioline). Quantitative real-time PCR was performed in a CFX Connect Real-Time Detection System (Bioline) to quantitate the relative levels of IFN and ISG mRNA in comparison to the house keeping gene RPLPO. Primers can be seen in Table 1.

**Table 1:** Primer table.

Name	Nucleotide Sequence
**RPLPO-FP**	5’-AGATGCAGCAGATCCGCAT-3’
**RPLPO-RP**	5’-GGATGGCCTTGCGCA-3’
**OAS-1-FP**	5’-TCCACCTGCTTCACAGAACTACA-3’
**OAS-1-RP**	5’-GGCGGATGAGGCTCTTGAG-3’
**IFIT-1-FP**	5’-AACTTAATGCAGGAAGAACATGACAA-3’
**IFIT-1-RP**	5’-CTGCCAGTCTGCCCATGTG-3’
**IFN-β-FP**	5’-TGTCAACATGACCAACAAGTGTCT-3’
**IFN-β-RP**	5’-GCAAGTTGTAGCTCATGGAAAGAG-3’
**IFN-λ1-FP**	5’-GGAAGAGTCACTCAAGCTGAAAAAC-3’
**IFN-λ1-RP**	5’-AGAAGCCTCAGGTCCCAATTC-3’
**Viperin-FP**	5’-GTGAGCAATGGAAGCCTGATC-3’
**Viperin-RP**	5’-GCTGTCACAGGAGATAGCGAGAA-3’

### Western blot for LC3-b

Huh-7 cells were given low serum media for up to 72 h before cells were lysed and lysates subjected to SDS-PAGE. Proteins were transferred to nitrocellulose membranes and probed for LC3-b (Rabbit anti-LC3 antibody, 1/1000 (Cell Signalling)) with detection of complexes with goat-anti-rabbit-HRP conjugate (Sigma) and chemiluminescence. Protein loading was normalised by re-probing filters for β-actin (anti-rabbit β-actin, 1/10,000, (Sigma)). Membranes were scanned using an Amersham 600 chemiluminescent imager. Densitometry to quantify western blot bands was performed using ImageJ software (http://imagej.nih.gov/ij/), provided in the public domain by the National Institutes of Health, Bethesda, MD, USA). All bands were normalized to β-actin and graphed as LC3I/II.

### Dual luciferase reporter assay

Luciferase experiments were performed essentially as previously described [18]. Huh-7 and/or HeLa cells were seeded at 7 × 10^4^ in 12 well plates, 24 hours prior to transient transfection using Viafect (Promega) with 1μg pISRE-firefly luciferase in combination with 10 ng of the constitutively Renilla expressing plasmid pRL-TK. Twenty-four hours following transfection, cells were stimulated with IFN-β (1000 U/ml) for 5 hours. Input renilla luciferase and ISRE driven firefly luciferase were measured at 5 hrs post IFN-β stimulation using the Stop and Glo dual-luciferase kit (Promega) on a CLARIOstar plate reader. All experiments were performed in at least triplicate.

### Viral infections and the use of viral mimics

Cells were infected at 50 HA units/ml of Sendai virus, in a volume of 300 μl per well of a 12 well culture plate. Cells were then washed with PBS three times before being re-incubated in their respective culture media. At the indicated time points post infection (pi), cells were harvested for RNA as described earlier, and real-time PCR was performed utilising primers outlined in Table 1. In order to assess a cellular response to the viral replication intermediates dsRNA and dsDNA the viral mimics, Poly dA:dT and poly I:C orpoly I:C rhodamine (Invivogen, CA,) were transfected into cells using DMRIE-C reagent (Life Technologies, AUS) as per manufacturer’s instructions at a concentration of 2 μg/ml.

### Staining and microscopy

Oil red O staining was performed on cells that were fixed in 10% formalin for 5 minutes, followed by a 1 minute wash in ddH_2_O. Slides were rinsed with 60% isopropanol prior to staining with a filtered 5% Oil Red O solution for 30 minutes, and subsequent rinsing with 60% isopropanol. Bodipy staining was performed as previously [19], with nucleic acid counter staining performed using DAPI (Sigma). All slides were visualised using a Nikon T*i-*E inverted microscope (Nikon, Tokyo, Japan); Quantification of mean fluorescence intensity was performed with defined regions of interest (ROI), using NIS Elements AR v.3.22, and exported to excel for further analysis. Data was normalised by background signal subtraction, and user defined ROI’s accounted for cell counts.

### Statistical analysis

Results are expressed as mean ± S.E. Student’s t test was used for statistical analysis. p < 0.05 was considered to be significant. All statistical analysis was performed using Prism 6 (GraphPad Software). All experiments were performed in at least triplicate.

## Results

### Media serum reduction reduces LD content without inducing autophagy in HeLa or Huh-7 cells

Reducing the FCS in cell culture media is a widely used method in the literature to decrease the LD mass in cells [20–24]. This method has been used with FCS concentrations ranging from 0.05–5% [25, 26], 2–5% [27] or as low as 0.05% [28]. The time frame for serum starvation is equally varied and can include timeframes between 15 min up to several weeks [29].

**Fig 1:**
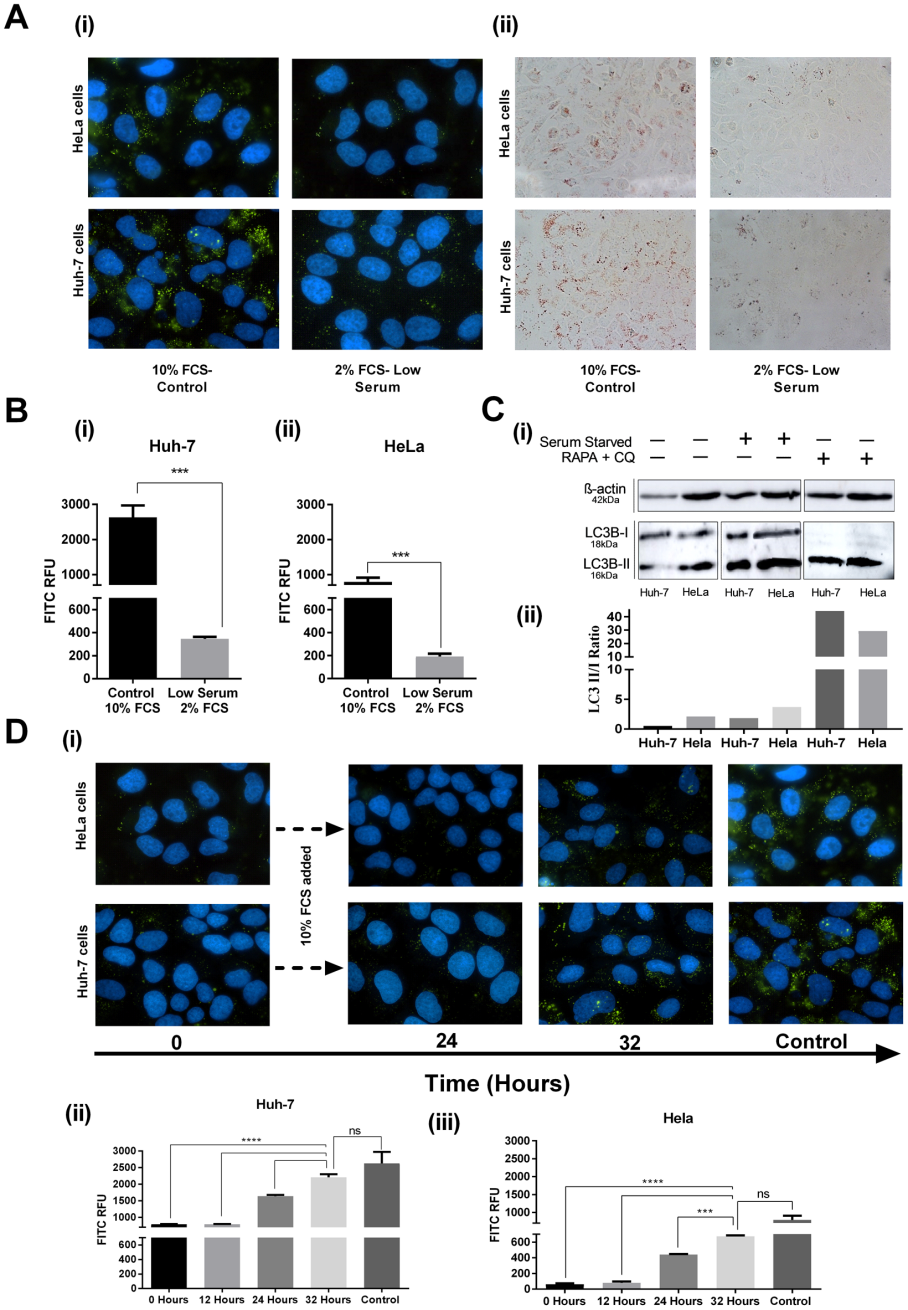
Lipid droplet mass is reduced by using lowered serum media, with little effect on their biogenesis. **A.** Reduced serum (10% to 2% FCS) was applied to Huh-7 and HeLa cells for 48 hrs. Bodipy 505/515 (i) and Oil Red O (ii) were used to stain neutral lipid in both Huh-7 and HeLa cells. Images of fixed cells were captured using a Nikon Eclipse Ti-E microscope at 20X and 40X magnification respectively. DAPI counterstaining was also used to visualise cell nuclei. **B.** Following visualisation of bodipy stained cells, quantification of fluorescence intensity was performed using NIS Elements AR v.3.22. ***p < 0.001 **C.** Cells were grown in low serum conditions (2% FCS) for 72 hrs. Rapamycin (RAPA) and Chloroquine (CQ) were used as a positive control for the induction of autophagy. Membranes were probed with anti-LC3 specific antibody and anti-rabbit HRP. Membranes scanned using Amersham 600 chemiluminescence imager (i); Densitometry was performed using Image J analysis (ii) **D.** LD number was reduced in Huh-7 and HeLa cells by reducing FCS in media to 2% for 48 hrs prior to experiment, representative by time 0. Cells were then returned to 10% FCS media at commencement for experiment, and fixed at the indicated time points. Cells were stained with Bodipy 505/515 and DAPI prior to imaging using a Nikon Eclipse Ti-E microscope at 20X magnification (i), and subsequent image analysis using NIS elements (ii).

In order to reduce the lipid content in cells we utilised a final FCS concentration of 2% for both Huh-7 and HeLa cell lines, and determined the reduction in relative LD abundance over time. Cells were initially plated in reduced FCS media for 24, 48 and 72 hours, and as can be seen in Fig 1A, a 48 h incubation with 2% FCS media, displayed a marked reduction in LD content as evidenced by staining of neutral lipids with either Bodipy or Oil Red O (Fig 1A), and quantification of Bodipy fluorescent intensity (Fig 1B). The LD reduction in both cell lines at 24 hrs was not significant.. As some cells can induce autophagy when lipid mass is lost, we also examined the induction of autophagy by observing the ratio of LC3-I to LC3-II in both cell types. Neither Huh-7 nor Hela cells underwent autophagy when grown in 2% FCS for periods of 72 hrs (Fig 1C, in comparison to the positive autophagy control, which displays a loss of LC3-I when treated with Rapamycin, in combination with the lysosomal inhibitor Chloroquine (Fig 1B) [30, 31].

The ability for cells to regenerate lipids after starvation is well documented in the literature [32–34]. Although, the basic principles governing LD biology are still not well defined, the mechanisms involved in the formation of LDs, protein targeting to LDs and the consequences of their shrinkage are only now being described (As Reviewed in [35]). In order to carry out all experiments in 10% FCS media, following a period in reduced serum, we wanted to determine the length of time for lipid-droplet regeneration in Huh-7 and HeLa cells following a period of incubation in reduced FCS media. Following 48 hrs in 2% FCS media, cells were returned to 10% FCS media, and Bodipy staining to detect neutral lipids performed at 24 and 32 hrs. Cells were found to regain the majority of their lipid biomass by 32 hrs, as can be seen by the similarity of the staining intensity to the control cells (10% FCS) (Fig 1D).

### Lowered lipid content effects the cellular response to type I IFN

**Fig 2:**
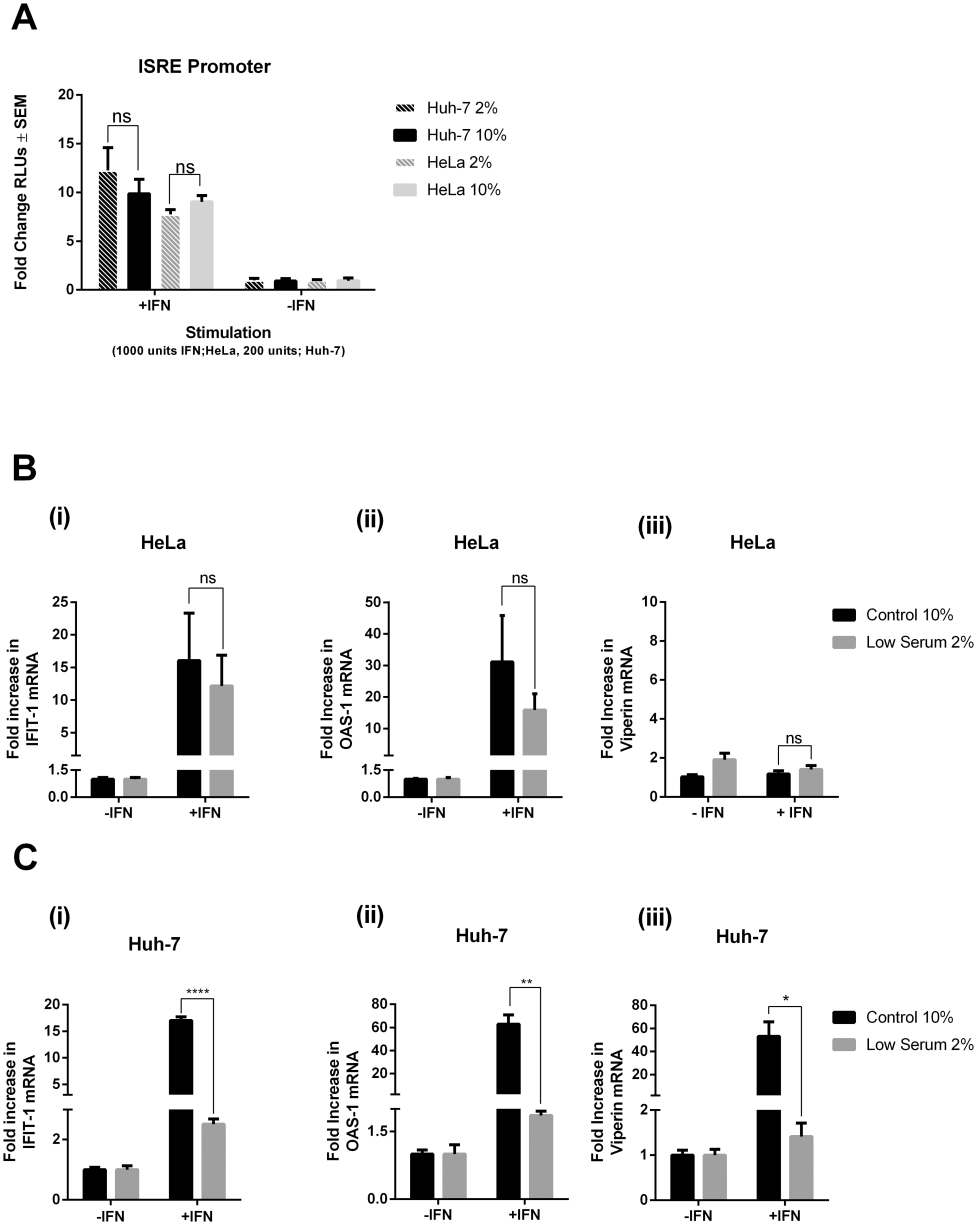
LD content alters the regulation of ISGs in a cell-type dependent manner. **A.** Huh-7 and HeLa cells were transiently transfected 24 hrs prior to stimulation with 500 ng/well of ISRE-Luc and 5 ng/well of pRL-TK in 12-well tissue culture plates. Luciferase measurements were taken 5 hrs post stimulation with IFN-β (ns = not statistically significant). **B.** HeLa and **C.** Huh-7 cells were pre-incubated in either low serum or control media 48 hrs prior to stimulation with 1000 units/ml of. IFN-β for the indicated times. RTq-PCR was used to quantify mRNA expression of (i) IFIT-1 (ii) OAS-1 and (iii) Viperin (ns = not statistically significant, *p<0.05, **p<0.01, ***p<0.001 ****p<0.0001).

We next wanted to assess whether a reduction in LD mass affected the ability of cells to respond to type I IFN, the predominant cytokine produced in response to viral infection of a host cell. Huh-7 and HeLa cells were plated in either low serum (2% FCS) or control serum (10% FCS) media, for 48 hours. All cells were placed into control serum media 30 minutes prior to transfection of an interferon stimulated response element (ISRE) promoter linked to the firefly luciferase gene, and stimulated 24 hrs post transfection with IFN-β for 5 hrs. As can be seen in Fig 2A, the relative increase in promoter activation in the control cells following IFN stimulation was similar to cells containing a reduced LD content, indicating that lowered LD mass does not impact ISRE activation following IFN-β stimulation. As not all ISGs are solely driven by ISRE activation, we also investigated the IFN driven regulation of a number of key known anti-viral ISGs that have previously been demonstrated to be upregulated independently of ISRE activation [36]. As can be seen in Fig 2B, no significant difference in IFIT-1 and OAS-1 mRNA expression was observed between control and low serum HeLa cells; with no regulation of viperin seen at all in these cells, even under interferon stimulation, as has been previously reported [37]. Conversely in Huh-7 cells, expression of all three ISGs was significantly impacted in the presence of reduced lipid content (Fig 2C). The mRNA for the ISGs, OAS-1 and viperin were not upregulated in the presence of IFN, in cells that were pre-treated to induce lowered LD mass (Fig 2C(ii) and 2C(iii)), and IFIT1 mRNA expression was only observed to increase by 2.5 fold (Fig 2C(i)). Collectively, IFIT1, OAS and viperin expression was found to be approximately 14, 60 and 52 fold reduced respectively (p < 0.05) in cells harbouring reduced LD numbers, in comparison to control treated cells. The differential results observed for the activation of ISRE’s and the induction of ISGs may indicate that lipid content is important in the activation of certain ISGs in a cell type specific manner.

### Lowered lipid droplet content does not effect the delivery of nucleic acids

**Fig 3:**
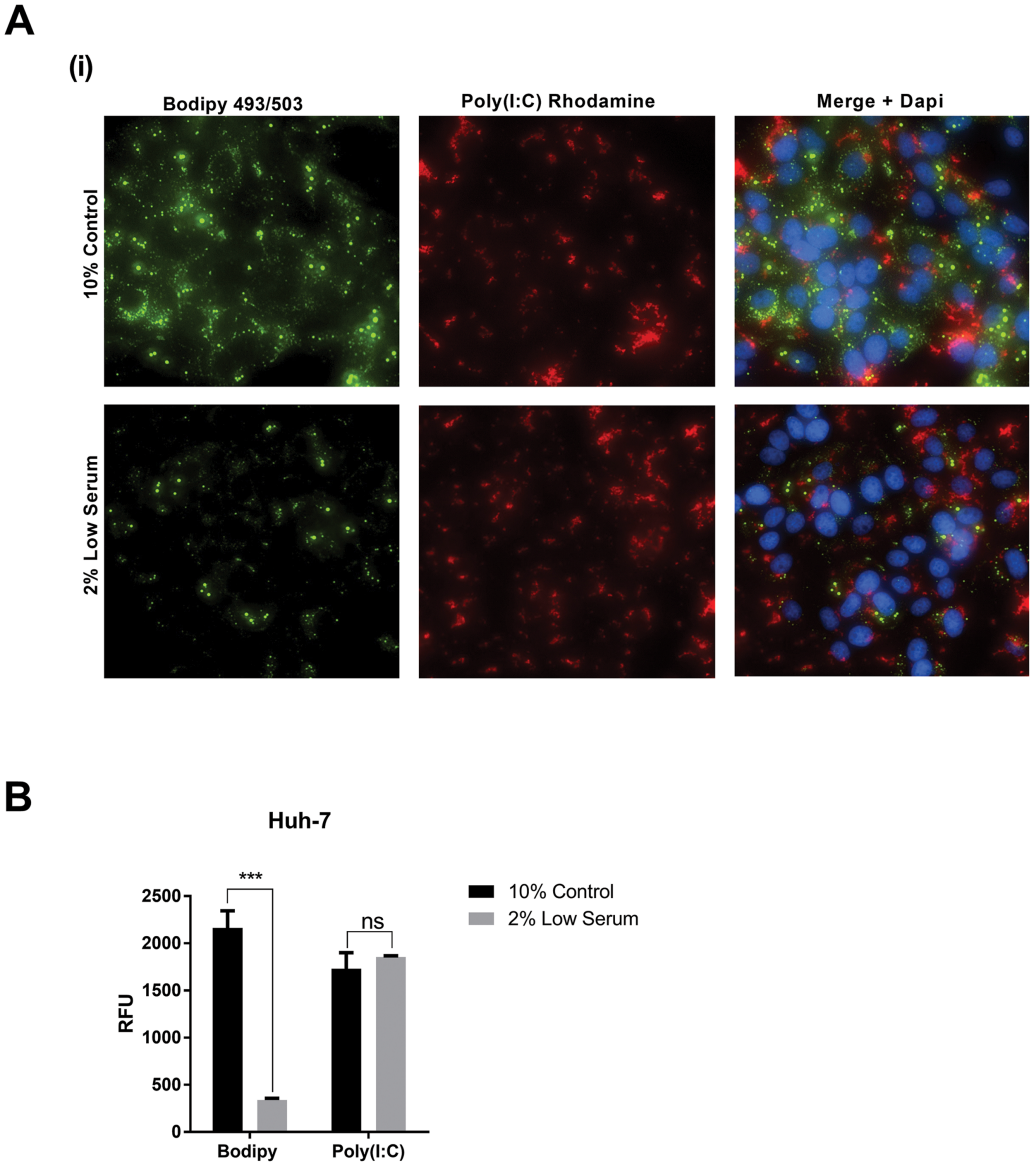
Lipid droplet content does not impact nucleic entry into Huh-7 cells. **A. A.** Huh-7 cells were either incubated in control media or 2% FCS media, 48 hrs prior to transfection with rhodamine conjugated poly I:C. **B.** Cells were imaged, using a Nikon T*i-*E inverted microscope and quantification of fluorescence intensity performed using NIS Elements AR v.3.22.

Prior to analysis of the host PRR signalling pathways in reduced LD cells, we first wanted to assess whether a lowered LD content would impact the delivery of viral mimics into cells. Utilising Huh-7 cells as a model cell line, we treated the cells as described above with lowered serum or control serum conditions for 48 hrs, prior to returning them to standard media. Cells were then transfected with a rhodamine conjugated poly I:C, and allowed to settle for 24 hours before imaging. As can be seen in Fig 3A and 3B, the ability of poly I:C to enter cells was not impacted in the reduced LD content cells, with fluorescent intensity of the rhodamine being equivalent in comparison to the much lowered intensity for Bodipy staining in the 2% serum treated cells, indicating the loss of LDs.

### Reduced LD content in cells reduces and delays the host response to dsDNA

**Fig 4:**
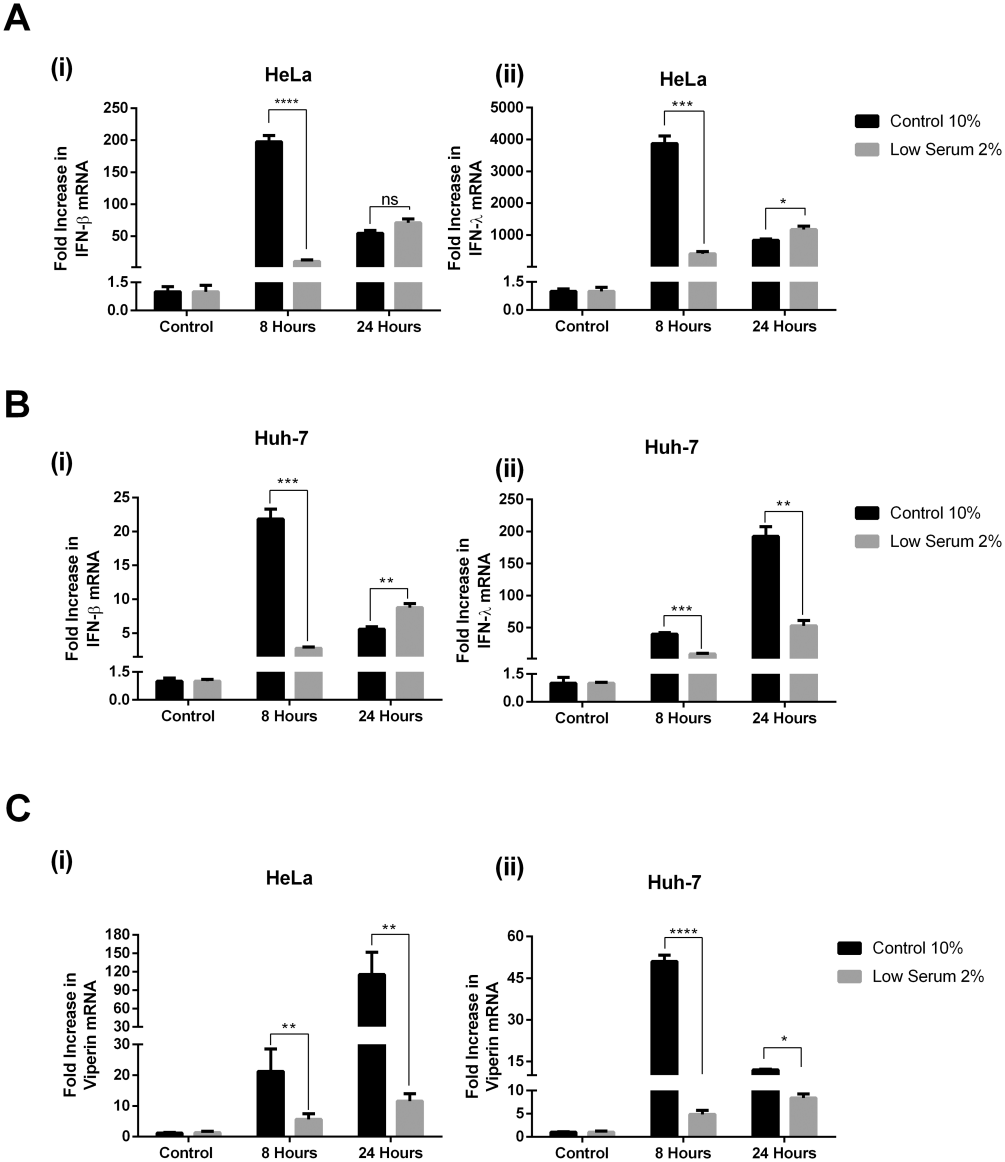
Reduced lipid droplet content in cells reduces and delays the host response to dsDNA. **A.** HeLa and **B.** Huh-7 cells were pre-incubated in either low serum or control media 48 hrs prior to transfection with 0.5 μg poly dA:dT per well for the indicated times. RTq-PCR was used to quantify mRNA expression of (i) IFN-β and (ii) IFN- λ or **C.** Viperin mRNA (ns = not statistically significant, *p<0.05, **p<0.01, ***p<0.001 ****p<0.0001).

Viral DNA in the cytosol of host infected cells is detected via a number of interferon induced cytosolic receptors, which all activate the common adaptor protein STING (stimulator of interferon genes), to ultimately induce the production of IFN [38]. To investigate the role LDs may play in this dsDNA signalling pathway, both control treated lowered LD Huh-7 and HeLa cells were stimulated with Poly (dA:dT) as a synthetic dsDNA viral mimic. To assess the ability of the cells to respond to dsDNA, via activation of IRF3, we examined expression of both IFN-β and IFN-λ at 8 and 24 hrs following stimulation, in conjunction with the upregulation of the ISG viperin. HeLa cells produced maximal amounts of both IFN-β and IFN-λ at 8 hours post dsDNA stimulation, however, cells with a reduced LD content displayed a 180 and 3500 fold decrease in IFN-β and IFN- λ production respectively (Fig 4A; p<0.0001 and p<0.001), with little upregulation of these IFNs at 8 hours following dsDNA stimulation in cells containing reduced LD content (Fig 4A). In contrast to the pattern of IFN upregulation observed in control cells, levels of both IFN’s were seen to increase in expression between 8 and 24 hrs in the cells with lowered LD content, whereas the control cells had a decrease in IFN expression between these two time points, indicating a lag in induction of these IFN’s in the presence of lowered LD content in HeLa cells (Fig 4A). A similar trend was observed in the Huh-7 cells for the upregulation of IFN-β in control cells compared to the LD reduced cells at an 8 and 24 hr time point following dsDNA stimulation (Fig 4B). Huh-7 cells reached maximum IFN- β expression earlier following dsDNA stimulation, in comparison to 24 hrs for the cells with the reduced LD content, also indicating a lag in the upregulation of IFN- β in the Huh-7 cells containing lowered LD numbers (Fig 4B). Interestingly, a similar trend was not observed for the expression of IFN- λ in the Huh-7 cells, where maximal cytokine expression was observed at 24 hrs regardless of the LD content in the cells; however, a reduction of IFN-λ expression was observed in Huh-7 cells with a reduced LD content at both 8 and 24 hrs following dsDNA stimulation (30 and 140 fold respectively; p<0.001, 0.01).

In order to determine if the reduction of IFN expression resulted in a decline in ISG expression we assessed the mRNA levels of the ISG viperin, which is known to be regulated via both type I and type III IFN [39]. As can be seen in Fig 4C, the relative reduction of both IFN- β and IFN- λ observed in both the HeLa and Huh-7 cells treated with dsDNA resulted in a decreased expression of viperin mRNA. HeLa cells showed significant upregulation of viperin mRNA at both 8 and 24 hrs post dsDNA treatment, however there was an approximate reduction in expression between the cells harbouring low levels of LDs and the control cells, of 4 and 10 fold respectively (p< 0.001). Huh-7 cells with a reduced LD content also displayed a small but significant increase in viperin mRNA at both 8 and 24 hrs following dsDNA stimulation (approximately 5 and 8 fold respectively; p < 0.05 and p<0.01), however, this equated to an approximate 12 and 1.5 fold decrease from the upregulation of viperin mRNA observed at these time points in the control cells (p<0.0001 and p<0.05). Interestingly, as viperin is known to not be driven by type I IFN in HeLa cells, and therefore presumably also type III IFN, (see Fig 2B, [37]), it is likely that the upregulation of viperin in this instance is directly IRF3 or IRF1 regulated, as has been shown previously (Reviewed in [14])

### Lipid droplet content alters the response to dsRNA

**Fig 5:**
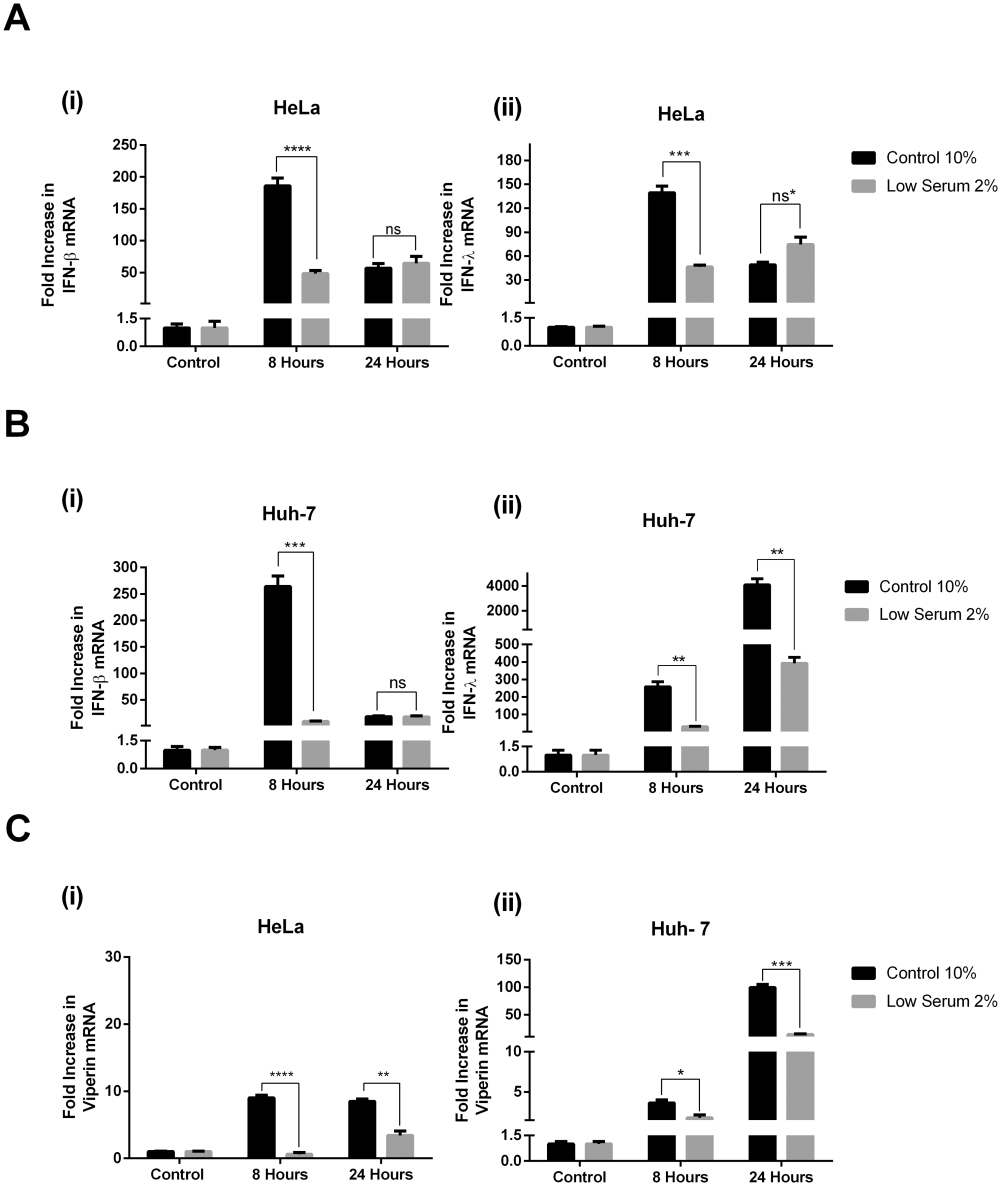
Reduced lipid droplet content in cells reduces and delays the host response to dsRNA. **A.** HeLa and **B.** Huh-7 cells were pre-incubated in either low serum or control media 48 hrs prior to transfection with 0.5 μg poly I:C per well for the indicated times. RTq-PCR was used to quantify mRNA expression of (i) IFN-β and (ii) IFN- λ or **C.** Viperin mRNA (ns = not statistically significant, *p<0.05, **p<0.01, ***p<0.001 ****p<0.0001).

RNA viruses are detected in the cytosol and during endosomal mediated entry through the RIG-I like and TLR3/7 pattern recognition receptor families of mammalian host cells (As reviewed in [40]). To investigate the role LDs may play in the RNA signalling pathways, we once again utilised the low serum media (2% FCS) conditions applied to both Huh-7 and HeLa cells. However, unlike HeLa cells, Huh-7 cells do not contain the dsRNA sensing receptor TLR3, and rely on their RIG-I like receptors to sense viral RNA [41]. Similar to what was observed following dsDNA stimulation, we also saw a reduction in IFN-β and IFN-λ expression in the LD reduced cells at 8 hours post dsRNA stimulation for both HeLa (137 and 193 fold respectively, p<0.001) and Huh-7 cells (254 and 229 fold respectively, p<0.01) (Fig 5A and 5B). In respect to IFN- β production, there was no difference in the ability of either control or LD reduced cells to enhance production in mRNA of this cytokine at 24 hours post dsRNA stimulation, however, the level of expression at this time point represented a drop for control cells, and a static position for both Huh-7 and HeLa cells with reduced LD mass (Fig 5A and 5B); an identical pattern was also observed for IFN- λ induction in both control and lipid reduced HeLa cells. In contrast, IFN-λ expression in the Huh-7 cells was at its highest at 24 hrs post dsRNA stimulation regardless of the cell treatments, however, expression was much reduced in cells with a lowered LD content at both 8 and 24 hours post stimulation (approximately 9 and 10 fold respectively, p<0.01); in a similar manner to what was seen for the dsDNA stimulation of these cells (Figs 4B and 5B).

As above, to determine if the reduction of IFN expression during dsRNA stimulation of LD depleted cells resulted in a decline in ISG expression we assessed the mRNA levels of the ISG viperin. Viperin mRNA expression induced by dsRNA was functionally reduced in the presence of a lowered LD content in both HeLa and Huh-7 cells (Fig 5C). HeLa cells displayed a marked reduction and a delayed induction of viperin mRNA in cells with a lowered LD content compared to control cells; while Huh-7 cells displayed a small 3-fold decrease in LD depleted cells compared with control cells at 8 hours post stimulation (*p<*0.05), with a much larger 70-fold decrease at 24 hrs post stimulation in the low serum cells compared to the control (*p<*0.001), which is converse to what was observed for dsDNA stimulation. This may indicate that LDs may have a larger role to play at early time points in the context of dsDNA stimulation of the ISG viperin, in contrast to later time points for induction of viperin via dsRNA (Figs 5C and 4C).

### Lowered lipid droplet content reduces the host response to SeV infection

**Fig 6:**
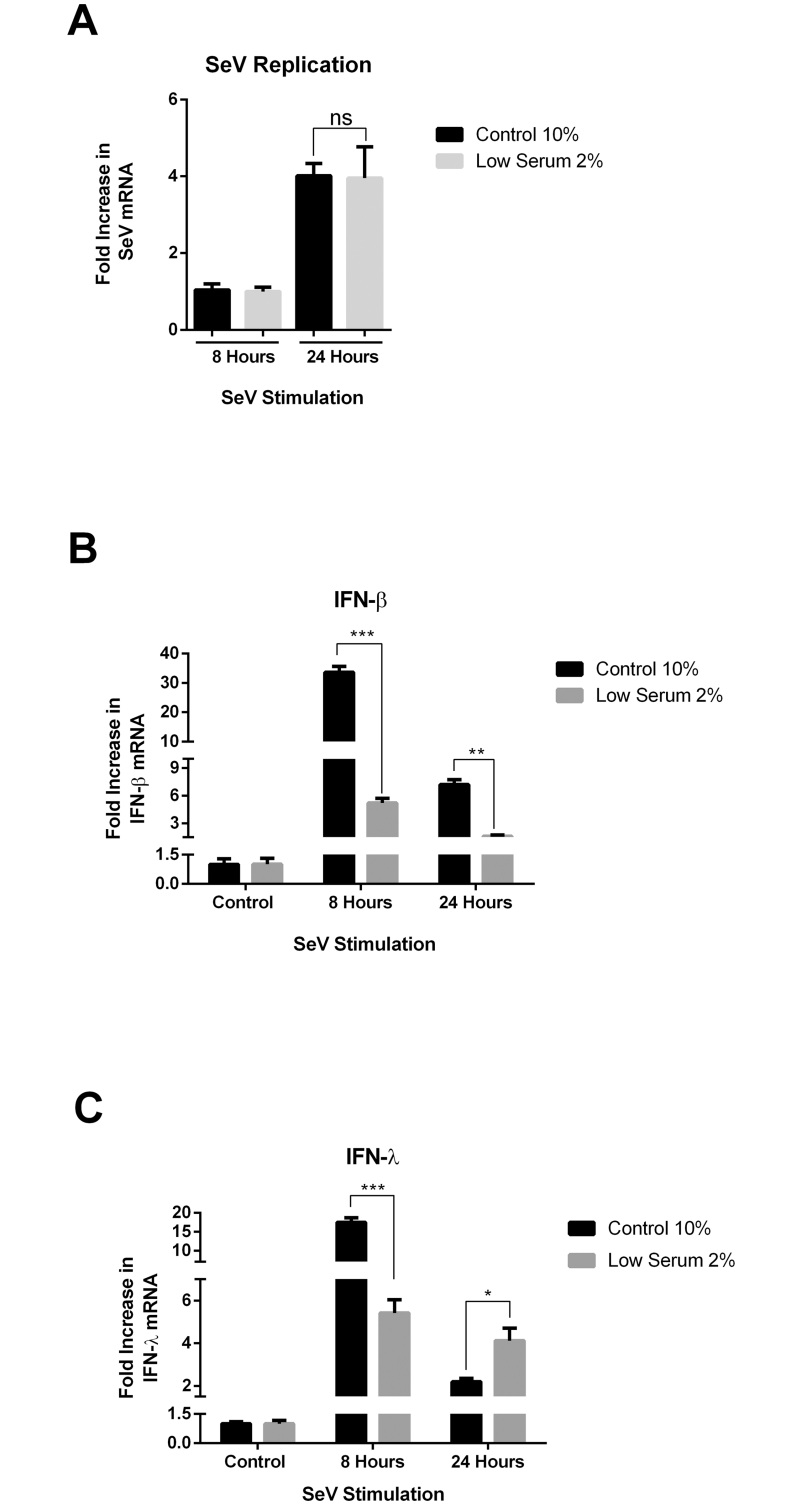
Lipid droplet content alters the host cell response to sendai virus. Hela cells were pre-incubated in either low serum or control media for 48 hrs prior to infection with 50 HAU/ ml SeV. Following 8 and 24 hrs infections of SeV, RTq-PCR was performed to quantitate mRNA expression of **A.** IFN-β and **B.** IFN-λ. (*, *p<* 0.05; **, *p<* 0.01; ***, *p<* 0.001).

We next wanted to examine the effects of LD reduction in a host cell response to a viral infection. Hela cells, known to have functional TLR3 and RIG-I [42] innate PRRs, were infected with the −ve sense ssRNA virus, sendai virus (SeV), and the upregulation of IFN mRNA examined as various time points post infection. Firstly we examined the ability of sendai virus to replicate in both control cells, and cells with a reduced LD content. Neither cellular entry, or replication of sendai virus differed between the two treated cell types, with no significant difference seen in viral RNA at either 8 or 24 hrs post infection (Fig 6A). However, despite no change in viral replication between the control and LD reduced cell lines, we saw a significant increase in the expression of both IFN-β and IFN-λ at 8 hrs post infection, with a marked increase in expression observed in the control cells versus the LD reduced cells (30 fold and 10 fold respectively, *p<*0.001, Fig 5B and 5C). Small, but significant differences were observed in both type I and III IFN responses at 24 hours post viral infection between the control cells and the LD reduced cells; however, interestingly the cells stimulated with SeV compared to the viral mimic of dsRNA did not show the delayed IFN response, and in both cases the 24 hr IFN response was less than that at 8 hours post infection (Figs 5A and 6).

## Discussion

Chronic and newly emerging viral infections contribute to growing human morbidity and mortality which threatens global health (Reviewed in [43]). The innate immune response, particularly the induction of IFN and ISGs, collectively establishes an antiviral state in the host, playing a significant role in limiting viral infections. The role of membrane-bound organelles as signalling platforms for the activation of key adaptor molecules within these innate signalling events has only recently gained attention [3]. It has been proposed that these signalling platforms are responsible for recruiting and assembling key adaptor molecules to aid in PRR pathway activation, influencing the intensity or speed of signalling pathway activation (reviewed in [3]). Interestingly, there has only been one instance to date of LDs acting as platforms in innate signalling [16]. As this organelle is widely used in other signalling pathways as a platform [35, 44], it is likely that its full role in the early innate immune response to viral infection is still to be uncovered.

To examine the broader role of LD’s in the context of induction of an IFN response to a viral infection, we sought to firstly establish an *in vitro* model of reduced LD mass. Functional biogenesis LD genes such as Arf79F in *Aedes aegypti* and mammalian Perilipin 1 and 2 have been knocked down effectively using RNAi technology, and demonstrate the inhibition of LD biogenesis [13, 45]. However, the use of RNAi to reduce LD biogenesis gene transcription is not well documented in the literature and may have other biological ramifications that have been largely unexplored [46]. Consequently, we chose the common method of reducing FCS content in cell culture media to reduce LD mass *in vitro*. This method was found to be highly reproducible when cells were placed in reduced serum media for 48 hrs prior to experimentation, with a complete complementation of LDs returning within 32 hrs, with no harmful effects such as autophagy induced.

We initially examined the ability of a lowered LD content to impact the cellular response to IFN *in vitro*, by examining the activation of the ISRE promoter. However, we saw no changes in the response of either cell line to IFN in the presence of a lowered LD content, perhaps suggesting that LDs are not a generally integral organelle of the JAK/STAT pathway. To look at this in a more focused fashion, we examined the upregulation of individual ISGs known to be anti-viral effectors, in the presence of a reduction in LDs. Interestingly, a significant decrease in the ability of type I IFN to drive expression of these genes was observed in Huh-7 cells, but not in Hela cells *in vitro*, indicating that LDs may play a role in the IFN induction of ISGs in a cell type specific manner. Although the production of IFN is induced in essentially all cell types, the induction of some ISGs has been shown to be more cell-type specific [47]. The two cell lines utilised in this study contain different basal levels of LDs, and this can visualised in Fig 1A, where it can be seen that the hepatocyte cell line, Huh-7, has an abundance of LDs [46], in contrast to HeLa cells which do not contain plenteous amounts [48]. This difference in the natural LD occurrence in the two cell lines could provide a possible explanation for the differences in the observed ISG expression, however more studies will be required to fully elucidate the mechanisms at play.

In comparison to the potential role of the LD in the JAK/STAT pathway, the dsDNA and dsRNA PRR recognition pathways showed a clear difference in the upregulation of IFNs between reduced lipid content cells and control cells. A reduction in LD mass was found to negatively impact both the magnitude and the speed of the type I and III interferon response to dsRNA and dsDNA *in vitro*. The magnitude of the IFN response to dsDNA and dsRNA was most notable at 8 hrs, with a significantly impeded upregulation of both IFN-β and IFN-λ in both cell lines. Interestingly, in the Hela cells there was little to no difference in the production of type I and III IFN in response to either nucleic acid at 24 hrs, with the levels of each representing a near steady state between 8 and 24 hrs in the LD reduced cells, and a reduction in expression levels in the control cells; indicating that in the Hela cells, a reduction in LD content mainly impacts the magnitude of the IFN response to RNA and DNA viral mimics. This effect was also seen in the context of the RNA virus, SeV, where infection of Hela cells resulted in a significant reduction of both type I and III IFNs in the cells with a lowered LD content, despite viral load being the same (Fig 6). In contrast, in the Huh-7 cells, a delayed induction of dsDNA induced type I and III IFN was seen in the lowered LD content cells, with only a delayed reduction of IFN-λ in the dsRNA treated cells. Interestingly, Huh-7 cells appeared to continue to increase their expression of IFN-λ from 8 hrs to 24 hrs in the context of both dsRNA and dsDNA viral mimic stimulation, irrespective of the LD status of the cells, in comparison to the fast response of Hela cells. Hepatocytes have been shown previously to induce ISGs with different kinetics through type I and III IFNs, with type III IFN showing a greater and lengthened response. Induction of Type III IFN is also the main driver of an antiviral response to the RNA virus HCV in Huh-7 cells *in vitro* [49, 50].

Reduced LD content in cultured cells seemed to not only have a reduced innate response to the DNA and RNA viral mimics, but also demonstrated a delayed response, which was more pronounced in the Huh-7 cells. One potential explanation for this occurrence, is that LD biogenesis occurs concurrently with the stimulation time, therefore, if LDs were playing an important role in these pathways, the production of IFN would increase with the increase in LDs. If the LDs were acting as a platform in these pathways, we can assume that the reduced LD cells would not reach their innate response peak at the same time.

LDs have previously been shown to localise proteins such as viperin, an antiviral ISG that inhibits a broad spectrum of viruses (as reviewed in [14]), and a key molecule in the antiviral immune response to viral infection through augmentation of the TLR7/9 pathways [16]. It has been assumed for some time that LD’s may play a role in the inflammatory response to bacterial and parasitic infection, often increasing in number upon cellular infection via a TLR dependent mechanism (As review in [51]). More recently, viral infection of mosquitoes has been demonstrated to induce enhanced LD formation in the cells of the midgut. This accumulation of LD’s was mimicked via activation of the Toll and IMD pathways through dsRNA stimulation in the mosquito; perhaps indicating that this accumulation of LDs is an important innate response to viral infection of the mosquito [52]. There has been very limited work performed to date examining the role of LDs in the early innate response to viral infection, with much of the research dominated by the hijacking of LDs for the completion of the viral life cycle (As review in [53]).

In this study, we have demonstrated a reduced activation of both the dsDNA and dsRNA signalling pathways under reduced LD conditions. This work underpins the hypothesis that the LD is an important organelle in the context of certain innate immune signalling pathways by adding further data to the observation that the LD is vital in TLR7 and 9 signalling. Clearly more work is required to fully elucidate the full role of the LD in the context of the early innate immune response to viral infection, however recent work has indicated that the LD may also be another key organelle in the context of antiviral signalling cascades.

## Abbreviations

ISG: interferon stimulated gene
LD: Lipid droplet
MAM: mitochondrial associated membrane
PAMP: pattern associated molecular pattern
PRR: pattern recognition receptor
SeV: sendai virus
TLR: Toll Like Receptor
